# The relevance of cortical lesions in patients with multiple sclerosis

**DOI:** 10.1186/s12883-016-0718-9

**Published:** 2016-10-21

**Authors:** Olivia Geisseler, Tobias Pflugshaupt, Ladina Bezzola, Katja Reuter, David Weller, Bernhard Schuknecht, Peter Brugger, Michael Linnebank

**Affiliations:** 1 Department of Neurology, University Hospital Zurich, Frauenklinikstrasse 26, 8091 Zurich, Switzerland; 2 Department of Psychology, University of Zurich, Binzmühlestrasse 14/1, 8050 Zürich, Switzerland; 3 Neurology and Neurorehabilitation Center, Luzerner Kantonsspital/State Hospital, 6000 Lucerne 16, Switzerland; 4 URPP Dynamics of Healthy Aging, University of Zurich, Andreasstrasse 15/Box 2, 8050 Zurich, Switzerland; 5 Medizinisch Radiologisches Institut, Bahnhofplatz 3, 8001 Zurich, Switzerland; 6 Department of Neurology, Helios-Klinik Hagen-Ambrock, Ambrocker Weg 60, 58091 Hagen, Germany

**Keywords:** Multiple sclerosis, Cortical lesions, Memory

## Abstract

**Background:**

Recent studies suggest that cortical lesions in multiple sclerosis (MS) substantially contribute to clinical disease severity. The present study aimed at investigating clinical, neuroanatomical, and cognitive correlates of these cortical lesions with a novel approach, i.e. by comparing two samples of relapsing-remitting multiple sclerosis (RRMS) patients, one group with and the other without cortical lesions.

**Methods:**

High-resolution structural MRI was acquired from 42 RRMS patients and 43 controls (HC). The patient group was dichotomized based on the presence versus absence of DIR-hyperintense cortex-involving lesions, resulting in a cortical lesion group (CL, *n* = 32) and a non-cortical lesion group (nCL, *n* =10). Cognitive functioning was assessed in all participants with a comprehensive neuropsychological battery, covering mnestic, executive, and attentional functions.

**Results:**

Highest densities of cortical lesions in the CL group were observed in the bilateral parahippocampal gyrus. Relative to HC, patients with cortical lesions - but not those without - showed significant global cortical thinning and mnestic deficits. The two patient groups did not differ from each other regarding demographic and basic disease characteristics such as EDSS scores.

**Conclusion:**

The appearance of cortical lesions in MS patients is associated with cortical thinning as well as mnestic deficits, which might be key characteristics of a 'cortically dominant' MS subtype.

**Electronic supplementary material:**

The online version of this article (doi:10.1186/s12883-016-0718-9) contains supplementary material, which is available to authorized users.

## Background

Traditionally, multiple sclerosis (MS) is considered as an autoimmune inflammatory disorder [1], predominantly affecting the white matter of the central nervous system [2]. This view is based on the high sensitivity of conventional MRI sequences in the detection of white matter abnormalities in MS patients. Nevertheless, histopathologic analyses of demyelinated foci in the cerebral cortex of patients with MS - dating back to the beginning of the 20^th^ century [3] - gave first evidence for cortical involvement in MS. In the early 1960s, Brownell and Hughes described that 26 % of the MS lesions affected the gray matter [4]. However, the classical view of MS as a pure white matter pathology has not been overcome until the beginning of the 21^st^ century. Due to the introduction of more sensitive imaging techniques such as double inversion recovery (DIR), gray matter involvement of the MS pathology is now well established [5, 6]. By suppressing the signals form the cerebrospinal fluid and white matter, DIR sequences have a higher sensitivity compared to conventional MR sequences [4] and thus, have made a major contribution in detecting focal cortical lesions in MS. Although a recent study has shown that DIR can detect only a minority of cortical lesions in MS, the same study has also shown a significant correlation between the number of DIR-hyperintense cortical lesions on the one hand and the total number of cortical lesions in post-mortem histopathologic analysis on the other hand [7]. Moreover, DIR is currently considered the best wide-scale application pulse sequence for cortical lesion detection and offers high sensitivity, specificity and accuracy for the detection of gray matter lesions [8].

Multiple sclerosis is associated with a variety of symptoms that are responsible for functional impairment in affected patients. Besides physical disability, cognitive deficits – which were firstly described in the second half of the 19^th^ century [9] – are found in up to 70 % of patients with multiple sclerosis at both the earlier and later stages of the disease [10]. MS-related cognitive impairment can affect various aspects of cognition. Processing speed and episodic memory seem most commonly affected [11]. However, MS patients often exhibit significant deficits in executive functions too [12, 13].

The extended focus in MS research including gray matter pathology had important consequences for the study of the neural correlates of MS symptoms. In recent years, the contribution of DIR-hyperintense cortical lesions to functional loss is particularly highlighted. Cross-sectional and longitudinal studies have shown correlations between the number and/or volume of cortical lesions and cognitive [14, 15] or physical [16] impairment. Regional associations are also described, for instance between an accumulation of cortical lesions in mesio-temporal areas and impairments in episodic memory [12, 17].

However, these studies are not without limitations. For example, detailed cognitive investigation [14] or DIR sequences were lacking [15]. Here we try to overcome these shortcomings and apply a novel methodological approach to investigate the clinical relevance of cortical lesions in MS, i.e. by dichotomizing patients based on the presence or absence of cortical lesions. The resulting two subgroups were compared with regard to demography, cognition, fatigue, affective mood state, and several other established MRI markers of disease severity, for example T2 lesion load, third ventricle width, and global cortical thinning.

## Methods

### Participants

Forty-two patients with a diagnosis of relapsing-remitting multiple sclerosis (RRMS) according to the McDonald 2010 criteria [18] were recruited at the Multiple Sclerosis Centre of the University Hospital of Zurich. Patients with at least one DIR-hyperintense cortex-involving lesion were assigned to the cortical lesion group (CL group, *n* = 32), the remaining patients formed the non-cortical lesion group (nCL group, *n* = 10). All patients received immunomodulatory treatment - 30 with natalizumab, seven with beta-interferons, three with fingolimod, one with glatiramer acetat and one with dimethylfumarate. Exclusion criteria were a relapse or steroid-treatment during the last two months, current or past neurological disorders in addition to multiple sclerosis, and psychiatric disorders apart from MS-related depressive mood state. Moreover, none of the patients was affected by severe visual deficits or upper limb sensorimotor impairment that could hinder cognitive test performance. Furthermore, 43 age-, gender-, handedness- and education-matched healthy control persons (HC) without previous or present history of neurological or psychiatric diseases were included. Controls received financial compensation for their attendance.

### MRI data acquisition

The MR scan was performed within one month of the neurological and neuropsychological examinations. All images were acquired using a 1.5-T scanner (Siemens Magnetom Avanto™) equipped with a SQ-engine gradient (45 m/T/m @ 200 T/m/s), using a dedicated 32-channel head coil. No hardware upgrades of the scanner occurred during the study period. The following sequences were performed in all subjects: (1) 3D Double Inversion Recovery (DIR) (voxel size = 1.5 x 1.5 x 1.5 mm, slice thickness = 1.5 mm, repetition time = 7500 ms, echo time = 308 ms, inversion timing 1 = 3000 ms, inversion timing 2 = 450 ms); (2) 3D T1-weighted MPRAGE (voxel size = 1 x 1 x 1 mm, slice thickness = 1 mm, repetition time = 2420 ms, echo time = 4.18 ms), and (3) 3D FLAIR (voxel size = 0.9 x 0.9 x 2.0 mm, slice thickness = 2 mm, repetition time = 5000 ms, echo time = 342 ms, inversion time = 1800 ms).

### MRI post-processing and statistical analysis

The classification of cortical lesions was conducted according to the consensus recommendations of Geurts and colleagues [19]. Consequently, cortical lesions were defined as those lesions appearing hyperintense on DIR images compared to surrounding normal-appearing gray matter, entirely or partly located in the cortical gray matter and occupying at least three voxels. Juxtacortical lesions (lesions not entering, but neighboring the cortical mantle) were not scored. DIR-hyperintense lesions were identified and manually delineated with MRIcron (http://people.cas.sc.edu/rorden/mricron/index.html), which was further used to measure total cortical lesion volume. An experienced rater, supervised by a neuroradiologist, assessed all images. The same procedure was applied to FLAIR images in order to identify FLAIR-hyperintense lesions. We used Statistical Parametric Mapping (SPM8, http://www.fil.ion.ucl.ac.uk/spm/) to co-register and normalize the individual lesion maps to MNI (Montreal Neurological Institute) standard space according to the normalization procedure proposed by Crinion and colleagues [20]. Normalized binary lesion maps were than plotted with MRIcron onto a T1-weighted MNI template brain to create lesion overlap plots. Central brain atrophy was examined by measuring the width of the third ventricle (TVW) according to the procedure proposed by Benedict and colleagues [21]. Brain parenchymal fraction (BPF), calculated as the ratio of brain parenchymal tissue volume to the total intracranial volume, was used as a measurement of whole-brain atrophy. The calculation was performed with Jim software (Xinapse Systems Ltd., Northants (UK); http://www.xinapse.com). Global cortical thickness evaluation was performed with the semi-automated Freesurfer image analysis suite based on MPRAGE images, which is documented and freely available online (https://surfer.nmr.mgh.harvard.edu/). Further information and technical details of these procedures are described in prior publications [22, 23]. To detect possible misclassifications of white and gray matter due to multiple sclerosis lesions, all images were visually inspected after the white/gray matter segmentation. In two patients, a semi-automated correction of topological defects was required. We used the manual procedure of control points, which is implemented in the Freesurfer software package. No further lesion masking was needed in order to obtain accurate reconstructions of the pial and the white matter surfaces.

### Clinical and neuropsychological assessment

All patients underwent neurological status examination, including the Expanded Disability Status Scale (EDSS). Cognitive functions were assessed with a comprehensive battery of validated and standardized neuropsychological tests. To minimize the issue of multiple statistical testing, composite index scores were computed for mnestic, executive and attentional functions by averaging z-scores for all subtests of the corresponding function [24]. An overview of the indices is shown in Additional file 1. The mnestic index score was derived from the delayed free recall and recognition of a 15-item word list [25] as well as the delayed free recall and recognition of a previously copied complex geometric figure [26]. Phonemic-verbal [27] and figural fluency [28], response inhibition [29] and cognitive flexibility [29] formed the executive index. The attentional index was based on processing speed during color naming [29] as well as on reaction times from tasks measuring alertness and selective attention [30]. Moreover, participants had to complete a German version [31] of the CES-D Depression questionnaire [32] and the Würzburg Fatigue Inventory (WEIMuS) [33] to self-rate depressive symptoms as well as fatigue during the last week. Similar to other studies [34], cognitive reserve was examined with passive vocabulary knowledge (multiple choice word test) [35].

### Statistical analysis

Statistical analyses were performed with SPSS (IBM, Chicago, USA, Version, 21.0, https://www.ibm.com/marketplace/cloud/statistical-analysis-and-reporting). Unless otherwise stated, a *p*-level below 5 % was considered statistically significant. Assumptions for normality were tested for all continuous data with Kolmogorov-Smirnov tests. In case of normally distributed variables, a multivariate ANOVA was used to compare groups. Bonferroni correction was applied for post-hoc analyses. When variables were not normally distributed, non-parametric tests were applied (Mann–Whitney U, Kruskal-Wallis).

## Results

### Demographic and clinical variables

Demographic and clinical characteristics of the three groups are reported in Table 1. No between-group differences were found with regard to age, education, cognitive reserve and gender. Furthermore, EDSS, age at diagnosis, and disease duration did not differ significantly between CL and nCL patients.

**Table 1:** Demographic and disease characteristics of multiple sclerosis patients and healthy controls

	nCL group (*n* = 10), Mean (SD)	CL group (*n* = 32), Mean (SD)	Controls (*n* = 43), Mean (SD)	Test	*p-*value
Age, years	32.6 (9.98)	38.22 (7.2)	36.1 (8.29)	One-way ANOVA	0.155
Education, years	15.5 (3.1)	14.45 (3.2)	14.58 (2.4)	KW	0.408
Cognitive reserve^a^	30.0 (3.1)	30.94 (3.4)	30.70 (2.3)	KW	0.689
EDSS	2.0 (1.8)	2.8 (1.8)	–	MW-U	0.174
Age at diagnosis, years	26.7 (9.7)	30.65 (7.6)	–	*t*-test	0.185
Disease duration*,* years	65.5 (59.6)	85.31 (68.9)	–	MW-U	0.570

*Abbreviations*: *nCL* patients without cortical lesions, *CL* patients with cortical lesions, *one-way ANOVA* one-way analysis of variance, *EDSS* Expanded Disability Status Scale, *KW* Kruskal-Wallis, *MW-U* Mann–Whitney U

^a^Cognitive reserve was examined with passive vocabulary knowledge (multiple choice word test)

### MRI markers

**Fig. 1:**
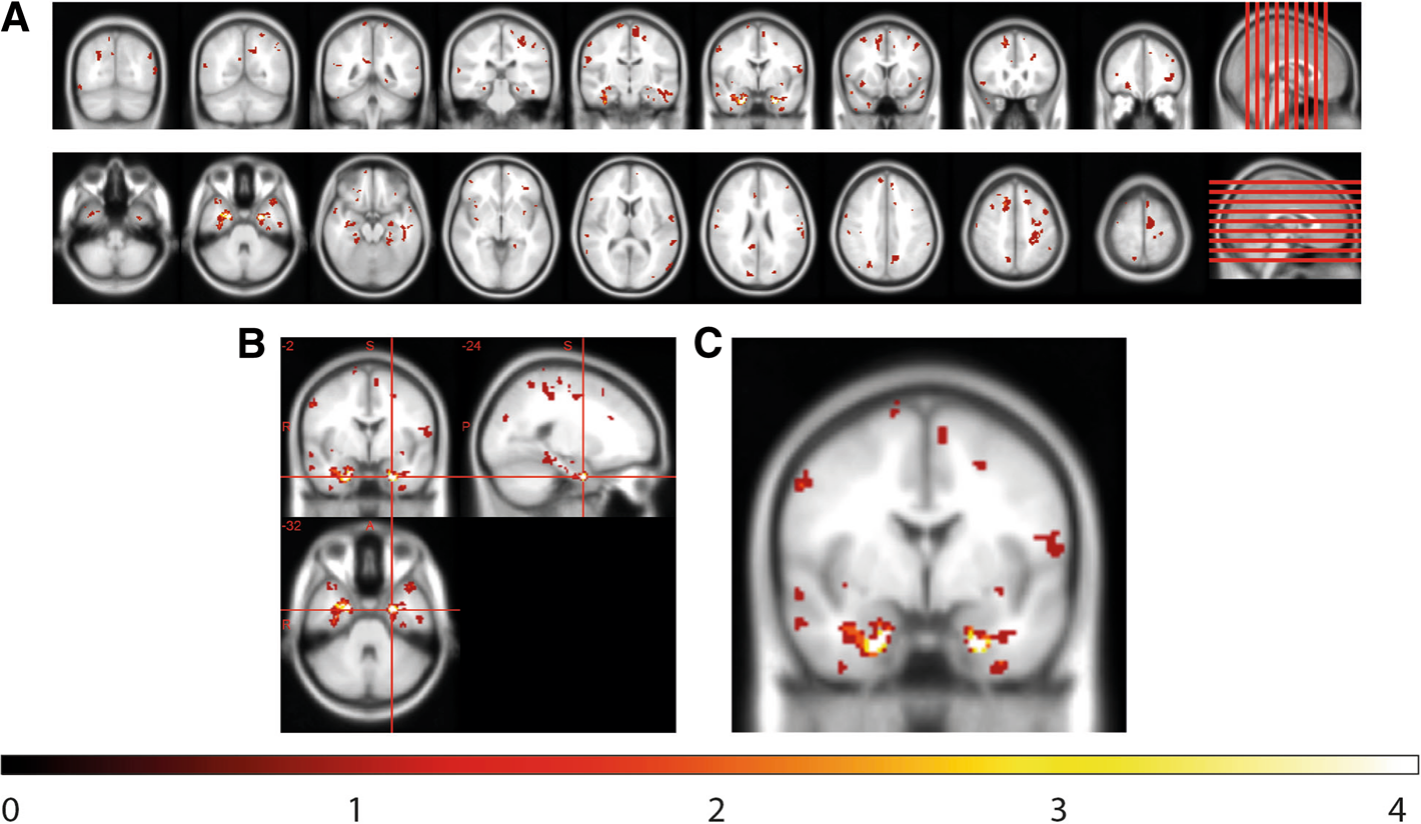
Spatial distribution of cortex-involving lesions in the CL patient group. Overlap plot based on all normalized cortex-involving lesions found in all CL patients. Lesion frequency across the sample is displayed for every depicted voxel. The bar indicates the number of patients showing damage to a particular voxel. **a**) Axial and coronal views of lesion frequency. **b**) and **c**) Highest lesion overlap was found in the bilateral parahippocampal gyrus (MNI coordinates in **b**). Image orientation follows the radiological convention (*right* on *left* side)

**Fig. 2:**
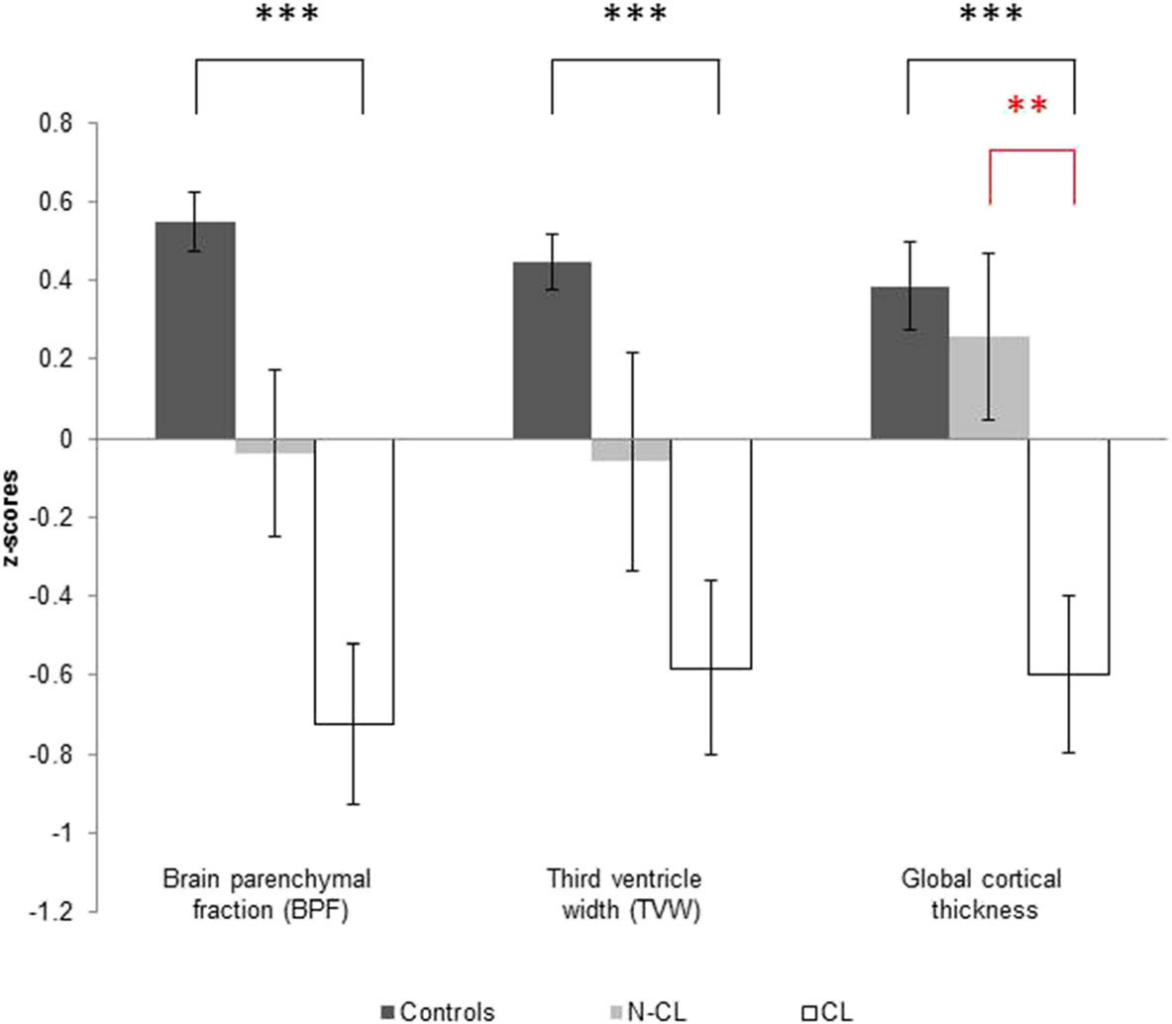
Atrophy measurements. Bars depict mean z-scores (and standard errors) of patients with (CL) and without cortex-involving (nCL) lesions as well as of healthy controls (HC). Note that the two patient groups differed only in global cortical thickness

We detected cortical lesions in 32 of 42 patients (76 %). The highest occurrence of cortical lesions was found bilaterally in the parahippocampal gyrus (Fig. 1). Twenty-five percent of the CL patients showed at least one cortical lesion in this region. An overview of the atrophy measurements in the three groups is given in Fig. 2. The three groups differed significantly from one another with regard to BPF (F(2) = 22.4, *p* < 0.001), TVW (H(2) = 20.9, *p* < 0.001) and global cortical thickness (F(2) = 11.6, *p* < 0.001). Bonferroni-corrected post-hoc analyses revealed a reduction of BPF in CL patients relative to HC (*p* < 0.001) as well as an enlarged TVW (*p* < 0.001). In contrast, the BPF and TVW values of nCL patients did not differ from those of CL patients or controls. Furthermore, Bonferroni-corrected post-hoc analyses revealed a reduction of global cortical thickness in CL patients compared to HC (*p* < 0.001) and nCL (*p* = 0.029) patients, while HC and nCL patients did not differ from one another. Regarding the FLAIR-hyperintense lesion volume, no significant difference (*p* = 0.064) was observed between the two patient groups.

### Cognitive test results, fatigue and depression questionnaires

**Fig. 3:**
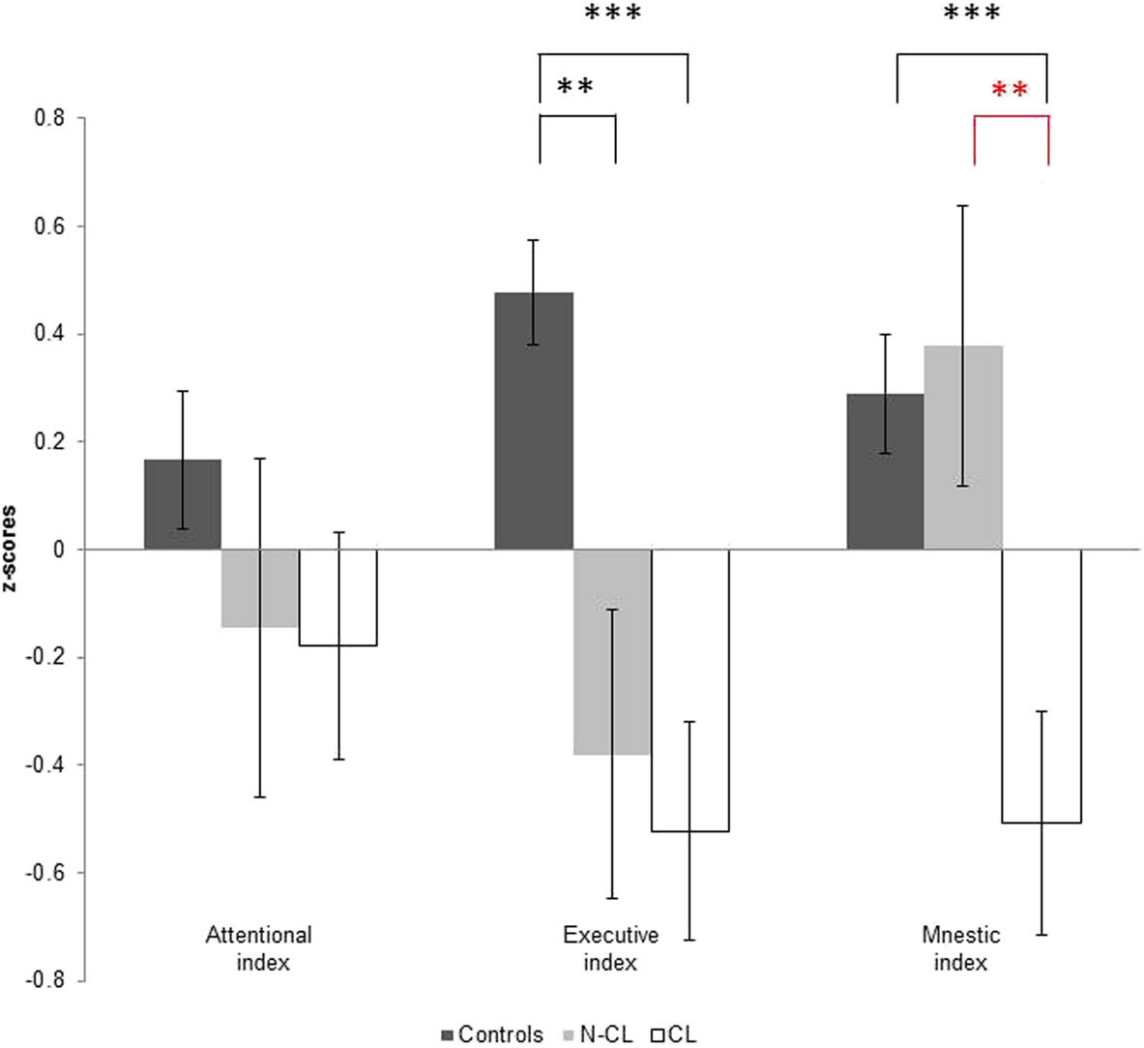
Indices of cognitive performance. *Bars* show mean z-scores (and standard errors) of patients with (CL) and without cortex-involving (nCL) lesions as well as of healthy controls (HC). Note that the two patient groups differed only in mnestic functioning

The attentional index did not differ significantly between the three groups (F(2) = 1.207, *p* = 0.304). In contrast, a group effect was observed for the executive index (H(2) = 22.476, *p* < 0.001). Both patient groups showed executive dysfunction compared to HC (CL vs. HC: Z(2) = −4.272, *p* < 0.001; nCL vs. HC: Z(2) = −3.197, *p* < 0.001), but did not differ from one another (Z(2) = −0.251, *p* = 0.805). Furthermore, there was a main group effect for mnestic functions (F(2) = 7.667, *p* < 0.001). Bonferroni-corrected post-hoc analyses revealed that the CL group performed significantly worse in memory tests than both nCL patients (*p = 0.*031) and HC (*p* < 0.001), whereas nCL patients did not differ from HC in this regard. Figure 3 depicts the three cognitive indices and corresponding differences between groups. According to supplemental analyses (summarized in Additional file 2) of the individual tests pooled in the memory index, differences between the two patient groups were most strongly pronounced in figural recognition. No main group effect was observed for depression (H(2) = 5.634, *p* = 0.060). However, both patient groups showed significantly enhanced fatigue scores compared to HC (CL vs. HC: Z(2) = −2.319, *p* = 0.020; nCL vs. HC: Z(2) = −2.102, *p* = 0.036), but did not differ from one another in this regard (Z(2) = −0.445, *p* = 0.673).

## Discussion

The impact of gray matter pathology, in particular that of gray matter lesions, on cognitive and physical functioning in MS patients has been discussed for many years. Here we highlight the clinical relevance of DIR-hyperintense cortical lesions in patients with RRMS. The most intriguing finding of the present study was that patients with - compared to those without visible cortical lesions - differed from each other in global cortical thickness and mnestic functions, whereas no differences between these two patient groups were observed regarding EDSS, age, age at diagnosis, disease duration, or non-mnestic cognitive functions. Moreover, patients without cortical lesions showed normal cortical thickness and mnestic functions, when compared with a group of healthy controls.

That a reduction of cortical thickness can occur in MS [36] - and that this thinning is related to global e.g. [37] and even specific cognitive impairment [12, 38] - has been found in previous studies. Similar to a recent finding [39], we show an association between MS-related cortical thinning and the presence of cortical MS lesions. It is known that gray matter pathology involves both inflammatory and degenerative mechanisms, but the relationship between the two remains unclear [6]. Gray matter atrophy might be the final step of several pathological processes, which could include cortical demyelination but also retrograde degeneration secondary to white matter lesions and, perhaps, primary neurodegeneration [40].

We detected DIR-hyperintense cortical lesions in 76 % of our RRMS patients, supporting the notion of a high prevalence of these lesions [16]. In further agreement with previous findings [41], we observed an uneven spatial distribution of cortical lesions over the cerebral cortex, with a prominent accumulation in memory-relevant mesiotemporal regions, particularly in the bilateral parahippocampal gyrus. While the importance of the hippocampus for memory function is known since the classical description of the patient H.M. in 1957 [42], parahippocampal involvement in memory functions was recognized only two decades ago [43]. Squire and Zola-Morgen identified the anatomical components of what is termed the medial temporal memory system [44]. By now it is well known that bilateral damage to the medial temporal lobe causes severe learning and memory impairments. This relationship has also been shown in MS patients. Learning and memory is the most frequently disrupted cognitive domain in MS, reported in 40–60 % of patients [45]. Moreover, Coebergh et al. [46] described a patient with acute memory impairment, associated with hippocampal and cortical lesions. Cortical lesions were also associated with cognitive decline in a group of 13 MS patients [17]. In this study, a significant correlation between hippocampal lesion load and visuospatial memory was observed. Based on the present and previous findings, we thus conclude that mesiotemporal cortical lesions are highly prevalent in RRMS patients and play a crucial role in the development of mnestic dysfunction.

The association we found between memory impairment and both cortical thinning as well as cortical lesions seems particularly intriguing: One might speculate that mnestic dysfunction in MS patients could indicate cortical involvement of the MS pathology in general. Related to this assumption, a rarely occurring variant of so-called 'cortical MS' has been described in previous studies [47, 48]. The condition was predominantly characterized by the presence of neurobehavioral symptoms (e.g. depression, apathy) and neuropsychological deficits (e.g. agraphia, anomia) suggesting cortical dysfunction. However, detailed and explicit imaging data for detecting cortical involvement (e.g. DIR) is missing in these studies. By approaching from the imaging side, our patient group with cortical involvement may reflect a different and somehow incomplete variant of cortical MS, as they showed distinct mnestic deficits in association with cortical involvement, but no other cortical symptoms such for example depression. Moreover, we found no patient with cortical lesions in the absence of subcortical lesions. In all patients, including those of the CL group, the majority of MS lesions was located subcortically. Taking this into account, we here propose that our CL group may represent a 'cortically dominant' subtype of MS. In these patients, pathophysiological processes might be different from those of patients without cortical involvement.

Beside the difference between the two patient groups in mnestic functions, both patient group showed executive deficits compared to the healthy control group. This finding is in line with previous studies that have shown significant executive dysfunction in MS patients [12, 13, 49].

Much research and clinical development in MS has focused on the inflammatory mechanisms of the disease. Meanwhile, multiple disease-modifying drugs (DMD) are available that target the inflammatory pathology of MS, in particular the development of new white matter lesions [50]. A recent study demonstrated that DMD - in particular IFN β-1a and glatiramer acetate - can reduce the accumulation of cortical lesions too [51]. In addition, Filippi and colleagues [52] showed that the presence of at least one cortical lesion is associated with a high risk of conversion from clinically isolated syndrome (CIS) to definite MS within a short period. Together with our results, these findings highlight the relevance of cortical lesions as a “target” in the development of new DMD, and to include cortical lesions as a primary outcome variable in disease and treatment monitoring.

This study is not without limitations. As already mentioned earlier, DIR represents only a limited snapshot of the real cortical pathology that is present in MS patients. The combination of DIR and a T1-weighted phase-sensitive inversion recovery (PSIR) sequence would substantially improve the sensitivity of detecting lesions present in the cortex of MS patients [53]. Moreover, the present analyses are limited due to the low MRI field (1.5 T) applied. It has been shown that high field (3 T) and ultra-high field (7 T) MRI systems deliver a higher detection rate of cortical lesions in vivo [54]. Finally, a further limitation of the present study is the relatively small and unbalanced sample size.

## Conclusion

In conclusion, the occurrence of cortical lesions in MS is clinically relevant insofar as it is associated with neurodegenerative cortical thinning and mnestic dysfunction. Although with today’s imaging techniques, it is only possible to visualize the 'tip of the iceberg' of cortical MS lesions [7, 55], further progress in detection algorithms can be expected and will likely improve our understanding of MS pathology, symptoms, and treatment.

## Additional files

Additional file 1: Neuropsychological tests summarized in the three cognitive indices. (XLS 25 kb)
Additional file 2: Detailed analyses of attentional, mnestic and executive functions (z-scores). (XLS 29 kb)

## Abbreviations

BPF: Brain parenchymal fraction
CL: Cortical lesion group
DIR: Double inversion recovery
EDSS: Expanded disability status scale
HC: Healthy controls
MRI: Magnetic resonance imaging
MS: Multiple sclerosis
nCL: Non-cortical lesion group
RRMS: Relapsing-remitting multiple sclerosis
TVW: Third ventricle width
